# Bioactive Potential of Post-Distillation Residue of *Clinopodium albanicum* (Griseb. ex K. Malý) Melnikov: Phytochemical Profiling, Antioxidant and Antimicrobial Activities with Molecular Docking Insights

**DOI:** 10.3390/plants15111748

**Published:** 2026-06-04

**Authors:** Dejan Stojković, Jelena Božunović, Biljana Filipović, Sergey Bolevich, Nikoleta Premović Valente, Marija Ivanov, Mladen Rajaković, Gokhan Zengin, Abdullahi Ibrahim Uba, Stefani Bolevich, Uroš Gašić, Marina Soković

**Affiliations:** 1 Institute for Biological Research “Siniša Stanković”—National Institute of the Republic of Serbia, University of Belgrade, Bulevar despota Stefana 142, 11108 Belgrade, Serbia; dejanbio@ibiss.bg.ac.rs (D.S.); jelena.boljevic@ibiss.bg.ac.rs (J.B.); biljana.nikolic@ibiss.bg.ac.rs (B.F.); nikoletapremovic@gmail.com (N.P.V.); marija.smiljkovic@ibiss.bg.ac.rs (M.I.); mladen.rajakovic@ibiss.bg.ac.rs (M.R.); uros.gasic@ibiss.bg.ac.rs (U.G.); 2 Department of Pathologic Physiology, First Moscow State Medical University I.M. Sechenov (Sechenov University), Trubetskaya Street, House 8, Building 2, 119991 Moscow, Russia; bolevich2011@yandex.ru (S.B.); alistra555@mail.ru (S.B.); 3 Department of Biology, Science Faculty, Selcuk University, Konya 42130, Türkiye; gokhanzengin@selcuk.edu.tr; 4 Department of Biostatistics and Medical Informatics, Faculty of Medicine, İstinye University, Istanbul 34396, Türkiye; abdullahi.iu2@gmail.com; 5 Department of Pharmacology, First Moscow State Medical University I.M. Sechenov (Sechenov University), Trubetskaya Street, House 8, Building 2, 119991 Moscow, Russia

**Keywords:** *Clinopodium albanicum*, hydrodistillation by-product, metabolomics, antioxidant activity, antimicrobial activity, antibiofilm, circular economy

## Abstract

The valorization of post-distillation by-products represents a key strategy within circular economy frameworks, particularly for medicinal and aromatic plants of the *Lamiaceae* family. This study investigates, for the first time, the chemical composition and biological potential of the liquid residue obtained after hydrodistillation of *Clinopodium albanicum* (Griseb. ex K.Malý) Melnikov, an endemic Balkan species. Untargeted LC–HRMS/MS analysis revealed a complex metabolomic profile dominated by hydroxycinnamic acid derivatives, including caffeoylquinic acids, alongside a diverse flavonoid fraction comprising quercetin, kaempferol, apigenin, and acacetin derivatives. The presence of sugars and organic acids further indicated a broad metabolic composition. The evaporated liquid residual extract exhibited strong antioxidant activity (DPPH: 32.54, ABTS: 27.80, FRAP: 35.95 mmol GAE/100 mg). Pronounced antibacterial activity was observed against both Gram-positive and Gram-negative bacteria, including *Staphylococcus aureus*, MRSA, *Listeria monocytogenes*, *Escherichia coli*, and *Pseudomonas aeruginosa* (MICs 0.5–1 mg/mL). Additionally, the extract demonstrated antifungal activity against *Candida auris* and *Candida parapsilosis*, as well as strong antibiofilm effects against *P. aeruginosa* (up to 95.52% inhibition). Molecular docking supported these findings, revealing strong binding affinities of key phenolics toward the bacterial targets FabI and D-Ala-D-Ala ligase. Overall, the results highlight the potential of this by-product for nutraceutical and pharmaceutical applications.

## 1. Introduction

The *Lamiaceae* family encompasses a diverse array of aromatic species that play an important role in traditional and natural medicine and have broad applications in the cosmetic, pharmaceutical, food, and agricultural industries [1]. These plants are rich in essential oils, whose distillation generates a significant amounts of by-products, including both solid and liquid residues [2]. In line with circular economy strategies, waste from the distillation process can serve as a cost-effective and sustainable resource for obtaining high-value bioactive compounds, as well as polysaccharides, which can be further used in various applications, such as food products, cosmetic preparations and innovative biomaterials [1,3]. Recent reports have highlighted solid post-distillation residues as valuable sources of bioactive metabolites, especially phenolic compounds [2,4,5]. During the extraction of essential oil, significant levels of non-volatile and heat-resistant compounds belonging to various groups of phenolic compounds remain in the solid residues. Phenolic extracts from post-distillation by-products of some commercially important species of Mediterranean flora (Greek oregano, rosemary, Greek sage, lemon balm and spearmint) exhibit strong antibacterial properties [5]. Luca et al. [2] demonstrated that the solid and liquid residues generated from essential oil extraction of three *Lamiaceae* species (thyme, oregano, and basil) are rich in bioactive metabolites, mainly phenolic compounds, with antioxidant and enzyme inhibitory properties.

The genus *Clinopodium* L. (*Lamiaceae*) currently comprises 192 species [6], including taxa recently reassigned from other genera as a result of comprehensive taxonomic revisions. Among these are members of section *Pseudomelissa*, formerly included in the polyphyletic genus *Micromeria* [7,8]. Species of *Clinopodium* are widely distributed across the globe and are characterized by a rich content of essential oils and diverse bioactive metabolites of pharmacological importance, including terpenoids, flavonoids, phenylpropanoids, lignans, and fatty acids [6]. Literature data indicate that *Clinopodium* metabolites exhibit diverse biological activities, including antimicrobial, antioxidant, anti-inflammatory, antitumour, hypoglycaemic, and insecticidal effects, supporting their medicinal, culinary, and biopesticidal applications [6,9].

*C. albanicum* (Griseb. ex K.Malý) Melnikov (syn. *Micromeria albanica* (K. Malý) Šilić; *Satureja albanica* Griseb. ex K. Malý), belonging to section *Pseudomelissa*, is an endemic plant of the Balkan Peninsula, distributed only in Albania and the Prizren area [10]. Recently, Matevski [11] discovered populations of *C. albanicum* in the western region of North Macedonia, specifically in the Demir Hisar area. Previous phytochemical investigations on this species have shown that the essential oil from the aerial parts of flowering plants is characterized by a high content of volatile ketones and epoxyketones [12]. Currently, the only data available in the literature regarding the biological potential of *C. albanicum* are from the study by Marinković et al. [10], which showed that the essential oil derived from the leaves of *C. albanicum* is a high-yield oil (0.88%) with strong dual antimicrobial activity. According to this study, the essential oil of *C. albanicum* exhibited the strongest antifungal activity when compared with the oils of *C. dalmaticum* (Benth.) Bräuchler & Heubl (syn. *M. dalmatica* Benth.) and *C. album* (Waldst. & Kit.) Bräuchler & Govaerts (syn. *M. thymifolia* (Scop.) Fritsch). In addition, oils from both *C. albanicum* and *C. dalmaticum* inhibited the growth of the highly resistant Gram-negative bacterium *Pseudomonas aeruginosa* [10].

In addition to the essential oil, phenolic compounds have also been studied in this species. Chemical profiling of flavone aglycones on the leaf surface of *C. albanicum* revealed the accumulation of significant amounts of external flavonoids of the 5,6-dihydroxy-7-methoxy type [13]. Acacetin glycosides were among the major flavonoids identified in methanolic leaf extracts of *C. albanicum* [14]. Dunkić et al. [15] found that four Balkan species from section *Pseudomellissa*, including *C. albanicum* (referred to as *C. serpyllifolium* in their study) possess high contents of total polyphenols, tannins, flavonoids, and phenolic acids.

However, information on the metabolic composition, antioxidant, and antimicrobial capacities of *C. albanicum* post-distillation by-products remains unknown. Particularly for endemic plants, these by-products can have unique localized chemical profiles with high pharmaceutical and nutraceutical potential [16]. Therefore, in the present study, lyophilized by-products generated during essential oil hydrodistillation of *C. albanicum* were characterized by untargeted liquid chromatography (LC)/Orbitrap mass spectrometry (MS). The bioactivity of the *C. albanicum* solid by-product was evaluated by in vitro antioxidant (metal-reducing and radical scavenging) and antibacterial (against Gram-positive and Gram-negative bacteria) tests. Additionally, an in silico interaction study of the major *C. albanicum* by-product compounds with the antimicrobial target enzymes was conducted to investigate their pharmacological potential.

## 2. Results and Discussion

To date, we have not found reports on the chemical composition or bioactive properties of *C. albanicum* by-products generated during essential oil hydrodistillation.

### 2.1. Chemical Analysis

**Figure 1:**
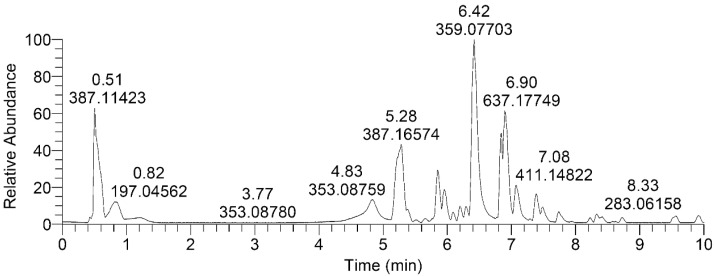
Relative abundance of detected compounds in the chromatogram.

*C. albanicum* can be described as a phenolic-rich species with a metabolome dominated by hydroxycinnamic acid derivatives and a broad accompanying flavonoid fraction, together with a notable contribution of primary and other specialized metabolites. In the sample, all annotated metabolites in Table 1 were detected, indicating a chemically complex extract rather than a narrow profile defined by only a few dominant constituents. The strongest analytical signal was assigned to rosmarinic acid (Figure 1), which clearly emerged as the principal metabolite of the sample. This finding is particularly important because rosmarinic acid is one of the most characteristic bioactive phenolics in *Lamiaceae* species and is frequently associated with antioxidant, anti-inflammatory, and antimicrobial activities. Its predominance suggests that the phenylpropanoid pathway leading to caffeic acid oligomers and esters is one of the major metabolic features of this plant material. Among the most abundant compounds were 4-*O*-caffeoylquinic acid and sagerinic acid, while salvianolic acid K, lithospermic acid, caffeoylshikimic acid, 4-*O*-feruloylquinic acid, 4-*O*-coumaroylquinic acid, ferulic acid, caffeic acid, *p*-coumaric acid, coumaric acid hexoside, caffeic acid hexoside, bis-caffeic acid hexoside, methylrosmarinic acid hexoside, and several yunnaneic acid derivatives were also present. Altogether, this set of compounds shows that the extract is not merely enriched in simple phenolic acids but contains an extended network of caffeic acid-derived conjugates and oligomeric phenolics. Such a pattern strongly supports the view that the phytochemical identity of the extract is centered on rosmarinic acid-related metabolism. This feature places the species within a chemically coherent *Lamiaceae*-type phenolic framework and provides a plausible basis for its biological activity.

The hydroxybenzoic acid fraction was present as a smaller but still relevant component of the metabolomics profile. This group included hydroxybenzoic acid hexoside, syringic acid hexoside, vanillic acid hexoside, gallic acid hexoside, dihydroxybenzoic acid hexoside, dihydroxybenzoic acid, vanillic acid, and hydroxybenzoic acid. These compounds indicate the presence of both glycosylated storage forms and free low-molecular-weight benzoic acid derivatives. Their contribution appears secondary compared with the hydroxycinnamic acid-rich fraction, but they still add to the overall phenolic character of the extract and may contribute to the antioxidant capacity of the sample. This fraction is considered part of the general (basal) phenolic background, while hydroxycinnamic acid derivatives are identified as the predominant class of specialized phenolic compounds.

The flavonoid profile of the *C. albanicum* by-product was also highly developed and chemically diverse. The glycosylated flavonoid fraction comprised quercetin 3,7-di-*O*-hexoside, apigenin 6,8-di-*C*-hexoside, quercetin 3-*O*-(6″-rhamnosyl)-hexoside, quercetin 3-*O*-hexoside, kaempferol 3-*O*-(6″-rhamnosyl)-hexoside, apigenin 7-*O*-(6″-rhamnosyl)-hexoside, kaempferol 3-*O*-hexoside, quercetin 3-*O*-(6″-acetyl)-hexoside, diosmetin 7-*O*-(6″-rhamnosyl)-hexoside, apigenin 7-*O*-hexoside, kaempferol 3-*O*-(6″-acetyl)-hexoside, and acacetin 7-*O*-(6″-rhamnosyl)-hexoside. Within this group, quercetin glycosides were especially prominent, with quercetin 3-*O*-(6″-rhamnosyl)-hexoside and quercetin 3-*O*-hexoside among the most intense flavonoid-related peaks in the sample. This suggests that flavonol glycosylation is an important metabolic feature in *C. albanicum*, probably reflecting the natural stabilization, storage, and compartmentalization of these metabolites in plant tissues. The occurrence of both rhamnosylated and acetylated derivatives further emphasizes the structural diversity of the flavonoid pool.

In addition to the glycosides, the sample also contained a substantial number of free flavonoid aglycones and methoxylated flavonoids, namely luteolin, quercetin, naringenin, apigenin, kaempferol, diosmetin, chrysin, acacetin, trihydroxy-dimethoxyflavone isomers, trihydroxy-trimethoxyflavone isomers, quercetin 3′,4′,7-trimethyl ether, and dihydroxy-tetramethoxyflavone. The presence of these compounds broadens the interpretation of the extract considerably. Rather than being composed mainly of highly polar glycosides, the *C. albanicum* by-product metabolomics profile also includes more lipophilic flavonoid constituents, especially methoxylated derivatives. These molecules can be important from a biological standpoint because methoxylation often affects membrane permeability, metabolic stability, and interaction with biological targets. The relatively high signal observed for trihydroxy-trimethoxyflavone isomer 1 and acacetin indicates that methoxylated flavonoids are not marginal constituents but are part of the characteristic chemical identity of the sample.

Another notable feature of the chemical profile is the strong contribution of non-phenolic and primary metabolites. Sucrose, quinic acid, malic acid, hexose, salvianic acid A, tuliposide B, maleic acid, tuberonic acid hexoside, benzoyl-glyceryl hexuronic acid, vinylphenyl-pentosyl-hexoside, syringaresinol hexoside, trihydroxy-octadecadienoic acid, and linoleic acid hydroperoxide were all detected. Particularly striking was the very high signal of tuberonic acid hexoside, which ranked among the most abundant metabolites in the sample, together with sucrose and quinic acid. This observation is interesting because it shows that the metabolic profile of the sample cannot be reduced to phenolics alone. The presence of abundant sugars and organic acids points to a matrix rich in central metabolites, while the occurrence of oxylipin-related compounds such as trihydroxy-octadecadienoic acid and linoleic acid hydroperoxide suggests active lipid-derived metabolism as well. Salvianic acid A is also noteworthy, since it belongs to the broader phenolic acid biosynthetic context and may complement the dominant rosmarinic acid-related chemistry. Altogether, these data indicate that the sample contains a complex blend of specialized and primary metabolites, which may be relevant when interpreting extract functionality, stability, and biological effects.

From the perspective of relative contribution, the chemical profile is mainly shaped by two large chemical domains. One is the hydroxycinnamic acid- and caffeic acid-derived fraction, and the other is the pool of other metabolites, including sugars, organic acids, and oxylipin-related compounds. The flavonoid glycosides form a substantial secondary fraction, whereas flavonoid aglycones and simple hydroxybenzoic acids contribute less to the total signal intensity. This means that the extract is chemically led by rosmarinic acid-related phenolics but supported by a broad metabolic background that may influence extraction behavior and bioactivity. In practical terms, rosmarinic acid, 4-*O*-caffeoylquinic acid, sagerinic acid, salvianolic acid K, and the major quercetin glycosides can be considered the key marker compounds of the sample.

Analytically, the sample annotations are strengthened by the very small mass deviations and by fragment ions that are chemically appropriate for the proposed structures. The hydroxycinnamic acid derivatives display the expected fragment patterns associated with caffeic acid, quinic acid, and related substructures, while the flavonoids show neutral losses and aglycone ions consistent with glycosylated quercetin, kaempferol, apigenin, diosmetin, and acacetin derivatives.

Taken together, the sample of *C. albanicum* is characterized by a chemically rich metabolite composition dominated by rosmarinic acid and other caffeic acid-derived conjugates, accompanied by a diverse flavonoid fraction composed of both glycosides and methoxylated aglycones, and further complemented by abundant sugars, organic acids, and oxylipin-related metabolites. The predominance of rosmarinic acid, caffeoylquinic acid derivatives, sagerinic acid, and salvianolic-type compounds indicates that phenylpropanoid metabolism is a central biosynthetic feature of the sample, while the substantial quercetin-, kaempferol-, apigenin-, diosmetin-, and acacetin-based flavonoid spectrum highlights the broad diversification of the flavonoid pathway. This phytochemical pattern supports the view that the sample is a phenolic-rich extract with strong potential for antioxidant and other bioactive properties and provides a robust chemical basis for further biological correlation studies.

### 2.2. Antioxidant Activity

The methanolic extract of *C. albanicum* by-product exhibited broad-spectrum antioxidant activity (Table 2), with radical scavenging potential in the DPPH assay of 32.54 mmol GAE/100 mg and 27.80 mmol GAE/100 mg in the ABTS assay. These findings are consistent with the FRAP results, which show strong reducing power for the *C. albanicum* extract (35.95 mmol GAE/100 mg).

The antioxidant potential of the methanolic extract of *C. albanicum* by-product, assessed by free radical scavenging (DPPH and ABTS) and reducing power (FRAP) assays, can be attributed to the predominance of hydroxycinnamic acids and flavonoids identified during qualitative chemical profiling in the present study. As illustrated in Figure 1, rosmarinic acid was the predominant compound identified in the methanolic extract of *C. albanicum* and is recognized for its potent antioxidant activity [21]. In addition, the high antioxidant activity of the by-product could be explained by the presence of antioxidant compounds, 4-*O*-caffeoylquinic acid and major quercetin glycosides [22,23], which were identified as key compounds in the methanolic extract. To the best of our knowledge, there is only one report on the antioxidant activity of hydrodistillation by-products from *Clinopodium* species [10]. Our results are in agreement with the significant antioxidant activity of the extract obtained from the hydrodistillation residual biomass of *C. vimineum,* which was dominated by a high content of rosmarinic acid [24]. According to this study, the hydroalcoholic extract obtained from plant material after distillation, as measured by the ABTS assay, showed the highest antioxidant activity (2400–2666 μmol Trolox^®^/g) compared with the essential oil and fresh material before distillation, due to a twofold increase in rosmarinic acid content. In the literature, several authors report significant antioxidant properties in other *Clinopodium* species and related *Micromeria* species. For example, the methanolic extract of *C. vulgare* L. subsp. *vulgare* L., rich in protocatechuic acid, catechin, chlorogenic acid, caffeic acid, ferulic acid, rosmarinic acid and apigenin, exhibited strong reducing power potential evaluated by the FRAP assay (87.25 ± mg TEs/g extract) [24]. In another study by Bektašević et al. [25], methanolic and aqueous extracts of *C. vulgare* were demonstrated to be good scavengers of free DPPH radicals. Vladimir Knežević et al. [26] established *M. croatica*, *M. juliana* and *M. thymifolia* as rich sources of antioxidant polyphenols, especially rosmarinic acid as the most important contributor to overall antioxidant effectiveness. Current results indicate that the by-product from the hydrodistillation process of aerial parts of *C. albanicum* contains phenolic compounds of particular interest, as they may be the key material basis for its pronounced antioxidant potential.

### 2.3. Antibacterial Activity

The results of the antibacterial activity are presented in Table 3. Among Gram-positive bacteria, *Listeria monocytogenes* strains LM 35152 and LM 19111 were the most susceptible, showing MICs of 0.5 mg/mL and MBCs of 1 mg/mL. Both *Staphylococcus aureus* ATCC and MRSA strains were inhibited at an MIC of 1 mg/mL and an MBC of 2 mg/mL. Among the Gram-negative bacteria, *Escherichia coli* was the most sensitive, with an MIC of 0.5 mg/mL and an MBC of 1 mg/mL, compared with *Pseudomonas aeruginosa* and *Salmonella typhimurium*, which displayed MICs of 1 mg/mL and MBCs of 2 mg/mL. These findings suggest that *C. albanicum* extract exerts a concentration-dependent bactericidal effect, with slightly higher efficacy against *L. monocytogenes* and *E. coli* than against the other tested strains. The antibacterial activity of the *C. albanicum* extract was lower than that of ampicillin, which showed MIC values in the μg/mL range (Table 3). The lower potency of the extract relative to the pure antibiotic is expected, considering that the extract represents a complex mixture of natural metabolites rather than a purified antimicrobial compound. The variation in susceptibility among the tested bacteria likely reflects differences in cell walls and membrane structures. Gram-positive bacteria lack an outer membrane, allowing bioactive compounds such as phenolics, flavonoids, and terpenoids to penetrate more easily and disrupt membranes or enzymes. Gram-negative bacteria possess an outer membrane with lipopolysaccharides, which can reduce compound penetration, partially explaining the higher MIC and MBC values for *P. aeruginosa* and *S. typhimurium* [27]. Previous studies have shown that species of the related genus *Micromeria* exhibit notable antibacterial activity. Essential oils from *M. fruticosa* have been reported to inhibit both Gram-positive and Gram-negative bacteria, likely due to their phenolic and terpenoid components [28]. Likewise, the essential oil of *M. thymifolia* not only suppresses bacterial growth but also interferes with quorum sensing in *P. aeruginosa*, a key pathway regulating virulence [27]. In addition, essential oil from *M. cilicica* demonstrates antimicrobial effects against pathogens such as *S. aureus* and *E. coli* [29], further highlighting the antibacterial potential of the genus.

### 2.4. Antifungal Activity

*C. albanicum* by-product exhibited a minimal inhibitory concentration (MIC) of 0.5 mg/mL and a minimal fungicidal concentration (MFC) of 1 mg/mL against *Candida auris*. These results indicate that the extract effectively inhibits the growth of this multidrug-resistant pathogen at relatively low concentrations, while a slightly higher concentration is required to achieve fungicidal activity. For *Candida parapsilosis*, the extract demonstrated stronger antifungal activity, with an MIC value of 0.25 mg/mL and an MFC value of 0.5 mg/mL. This suggests that *C. parapsilosis* is more susceptible to the *C. albanicum* extract than *C. auris* (Table 4). Compared with itraconazole, the *C. albanicum* extract exhibited lower antifungal potency, with MIC values of 0.25–0.50 mg/mL versus 0.003–0.006 mg/mL for the commercial antifungal. Despite this difference, the extract demonstrated clear fungicidal activity against both *Candida* species, including *C. auris*, as indicated by MFC/MIC ratios of 2. The antifungal activity observed for *C. albanicum* extract against *C. auris* may be attributed to the presence of bioactive phytochemicals characteristic of the genus *Micromeria*. These compounds can interfere with fungal growth through several mechanisms, such as disruption of the fungal cell membrane, inhibition of ergosterol biosynthesis, and interference with key metabolic pathways. Several studies have demonstrated that plant-derived compounds and essential oils exhibit significant inhibitory activity against *C. auris*, suggesting that natural products may represent promising sources of novel antifungal agents [30]. The antifungal effect of *C. parapsilosis* is often attributed to terpenoids and phenolic compounds that disrupt fungal cell membrane integrity, interfere with ergosterol synthesis, or induce oxidative stress, ultimately leading to growth inhibition or cell death [29,31].

### 2.5. Antibiofilm Activity

The antibiofilm activity of *C. albanicum* extracts was assessed using *Pseudomonas aeruginosa* as the test organism. The results of biofilm inhibition are presented in Table 5. The *C. albanicum* extract inhibited biofilm formation by 95.52% at the minimal inhibitory concentration (MIC), indicating a strong inhibitory effect. At ½ MIC, biofilm inhibition decreased slightly to 86.82%, while at ¼ MIC it reached 83.86%. These results show that the antibiofilm activity decreases moderately with decreasing concentration but remains high even at lower doses. At ⅛ MIC, inhibition dropped to 40.65%. Overall, the results indicate a dose-dependent relationship between extract concentration and biofilm inhibition. Species of *Micromeria*, as well as taxa formerly classified within this genus, such as *Clinopodium albanicum*, are rich sources of bioactive metabolites, including alcohols, aliphatic aldehydes, phenolic compounds (notably chlorogenic and ellagic acids), and flavonoids such as hesperetin, naringin, and quercetin [32]. These phytochemicals are known to possess antimicrobial and antibiofilm properties. Flavonoids mainly exert their antibiofilm activity through several mechanisms, including disruption of quorum-sensing (QS) systems, interference with global regulatory pathways, inhibition of bacterial adhesion, and suppression of extracellular polysaccharide synthesis, which is essential for biofilm matrix formation [33]. Furthermore, a study on *Micromeria thymifolia* demonstrated that its essential oil suppresses QS-regulated behaviors in *P. aeruginosa*, including swarming motility and pyocyanin production, both of which are important virulence factors involved in biofilm development [27]. Essential oil of *Micromeria nervosa* contains several bioactive compounds (α-pinene, caryophyllene oxide, and cadinene derivatives) that are known to interact with microbial metabolic pathways and enzymes, thereby reducing microbial growth and interfering with biofilm development [34].

### 2.6. Docking (Binding Energy) Scores

**Figure 2:**
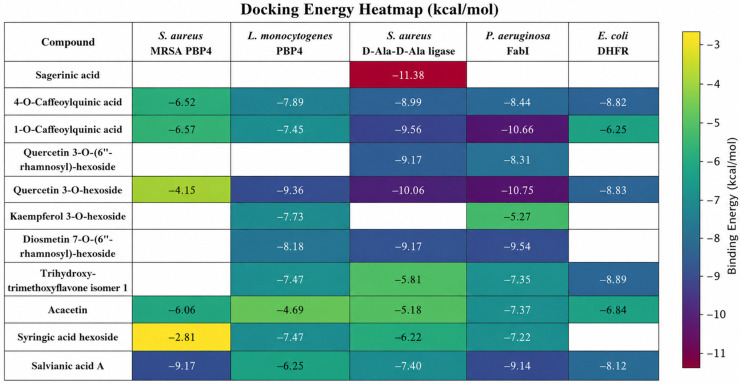
Heatmap representation of molecular docking binding energies (kcal/mol) of selected phytochemicals against key bacterial protein targets, including PBP4, D-Ala-D-Ala ligase, FabI, and DHFR. Color gradients indicate binding affinity, with more negative values (red) corresponding to stronger ligand–protein interactions and less negative values (yellow) indicating weaker binding. Missing values (blank cells) reflect unavailable docking results and should not be interpreted as weak binding.

The heatmap summarizes molecular docking binding energies (kcal/mol) of selected phytochemicals against multiple bacterial targets. Across the dataset, most compounds show moderate-to-strong interactions (approximately −6 to −11 kcal/mol), with clear variability depending on both ligand structure and protein target (Figure 2). Flavonoid derivatives such as quercetin glycosides demonstrate consistently strong binding across several targets. *Quercetin 3-O-hexoside* exhibits particularly high affinity, especially toward FabI (−10.75 kcal/mol), suggesting a favorable interaction profile with enzymes involved in fatty acid synthesis and cell wall biosynthesis. Similarly, caffeoylquinic acid derivatives display moderate-to-strong binding, with *1-O-caffeoylquinic acid* showing enhanced binding to FabI (−10.66 kcal/mol). Trihydroxy-trimethoxyflavone isomer 1 docked well into the active site of *Escherichia coli* DHFR (−8.89 kcal/mol). Salvianic acid A demonstrated moderate-to-strong binding, with docking energies ranging from approximately −6.25 to −9.14 kcal/mol, reflecting stable accommodation within multiple catalytic pockets. Validation of the molecular docking protocol by redocking the co-crystallized ligands into the active sites of their corresponding target proteins is presented in Supplementary Table S1.

#### 2.6.1. Protein–Ligand Interactions

The binding of 1-*O*-caffeoylquinic acid, quercetin 3-*O*-hexoside, trihydroxy-trimethoxyflavone isomer 1, and salvianic acid A to these targets was examined in comparison to the cocrystal ligand in each complex. These compounds formed stable hydrogen bonding, hydrophobic, and aromatic interactions with catalytically important residues involved in substrate recognition, cofactor stabilization, and inhibitor binding.

##### *Listeria monocytogenes* PBP4 with 1-O-Caffeoylquinic Acid

**Figure 3:**
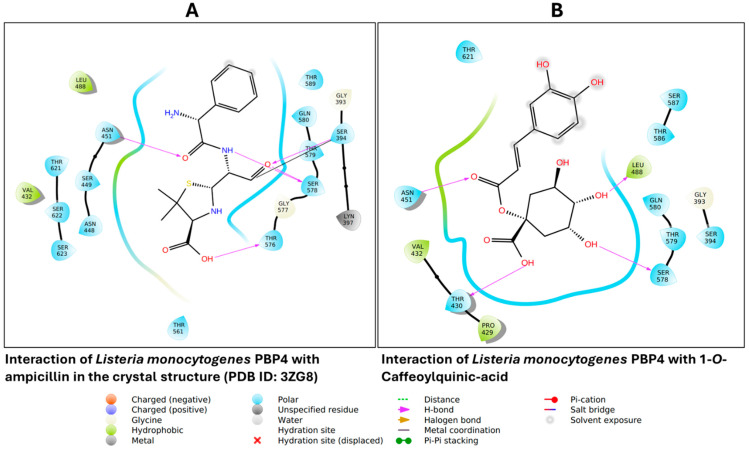
(**A**). Interaction of PBP4 from Listeria monocytogenes with ampicillin in the crystal structure (PDB ID: 3ZG8). (**B**). Interaction of *Listeria monocytogenes* PBP4 with 1-*O*-caffeoylquinic acid.

In the active site of *Listeria monocytogenes* PBP4, the cocrystal ligand ampicillin exhibited a relatively compact interaction pattern, mainly stabilized by hydrogen bonds with catalytic and nearby residues (Ser578, Thr576, and Asn451, and Ser394) (Figure 3A), consistent with the covalent acyl–enzyme binding mechanism of β-lactam antibiotics in PBP4 crystal structures [35].

Similarly, 1-*O*-caffeoylquinic acid formed hydrogen bonds with Ser578, Asp451, Gln391, and more residues (Figure 3B). Ser578 corresponds to the conserved catalytic serine of the transpeptidase active site in PBP4. This residue is directly involved in the acylation mechanism of β-lactam antibiotics such as ampicillin and is critical for peptidoglycan cross-linking activity [36]. Other important conserved active site residues include Gln580, a neighboring active site residue that mainly supports substrate recognition and stabilization [35]. These contacts anchor the ligand firmly within the binding cavity and highlight the importance of polar complementarity, which is widely recognized as a key factor in PBP4–ligand interactions [37].

Beyond these strong polar contacts, a couple of hydrophobic amino acids, including Val432 and Pro429, together with several van der Waals interactions, help accommodate the ligand scaffold. While these interactions are weaker than hydrogen bonds, they play an important role in maintaining ligand orientation and overall stability, consistent with general principles of protein–ligand recognition [38].

##### *Pseudomonas aeruginosa* FabI with Quercetin 3-*O*-Hexoside

**Figure 4:**
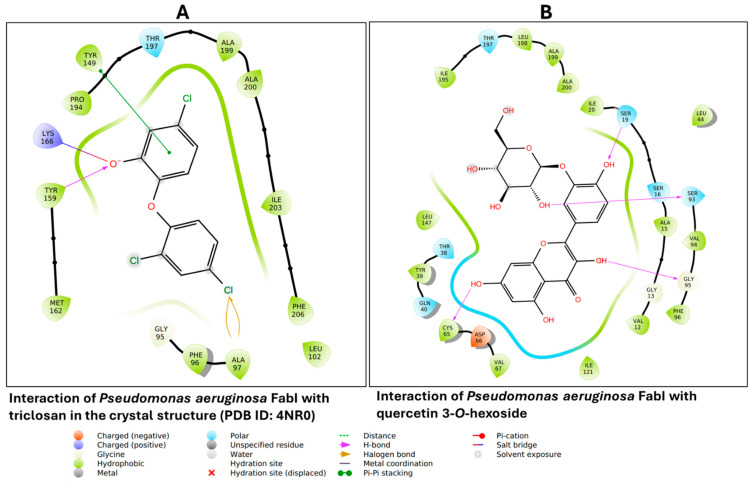
(**A**). Interaction of *Pseudomonas aeruginosa* FabI with triclosan in the crystal structure (PDB ID: 4NR0). (**B**). Interaction of *Pseudomonas aeruginosa* FabI with quercetin 3-*O*-hexoside.

Triclosan showed a mostly hydrophobic binding mode in the active pocket of *Pseudomonas aeruginosa* FabI, stabilized by a salt bridge with Lys166 and a hydrogen bond with Tyr159 (Figure 4A). This interaction pattern agrees with the known mode of action of triclosan, which forms a stable FabI–NAD+–triclosan ternary complex and inhibits fatty acid elongation [39].

In contrast, quercetin 3-*O*-hexoside, via its multiple hydroxyl groups, formed a more extensive hydrogen bonding network with catalytically and functionally important FabI residues, including Ser19, Se39, and Gly95 (Figure 4B), suggesting stable occupancy of both the catalytic and substrate recognition regions of the enzyme [40]. These interactions highlight the importance of polar complementarity, which is a common feature in FabI–inhibitor binding [41,42].

Around this polar core, a cluster of hydrophobic residues, including Val12, Val67, Val94, and several alanine residues, help accommodate the ligand’s aromatic rings. Aromatic residues such as Phe96 and Tyr39 further contribute to stabilizing the scaffold through non-specific contacts. While these interactions are weaker than hydrogen bonds, they play an important role in maintaining ligand orientation within the binding site [43].

##### *Escherichia coli* DHFR with Trihydroxy-Trimethoxyflavone Isomer 1

**Figure 5:**
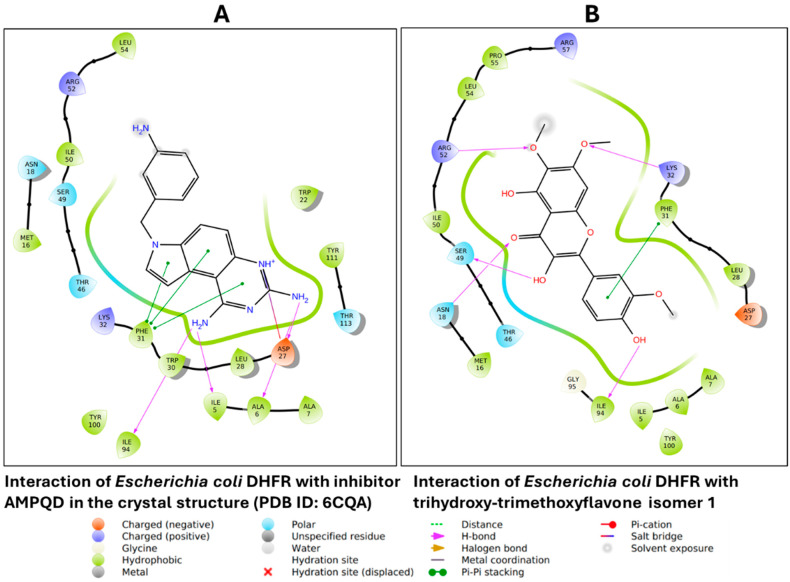
(**A**). Interaction of *Escherichia coli* DHFR with the inhibitor AMPQD in the crystal structure (PDB ID: 6CQA). (**B**). Interaction of *Escherichia coli* DHFR with trihydroxy-trimethoxyflavone isomer 1.

The binding of trihydroxy-trimethoxyflavone isomer 1 within the *Escherichia coli* DHFR active site is stabilized through a combination of hydrogen bonding and aromatic interactions (Figure 5). The ligand forms several key hydrogen bonds via its hydroxyl and methoxy oxygen atoms. Specifically, interactions are observed with Arg52 and Lys32, indicating favorable contacts between the ligand oxygen atoms and positively charged residues. Additional hydrogen bonding with Asn18 and Ser49 further contributes to anchoring the ligand within the binding pocket.

A distinctive feature of this complex is the presence of a π–π stacking interaction between the aromatic ring of the ligand and Phe31. This interaction likely enhances binding stability by aligning the ligand’s aromatic system with the phenyl side chain of Phe31, a feature commonly observed in DHFR–inhibitor complexes [44]. Surrounding hydrophobic residues further support ligand accommodation, helping to maintain the ligand’s orientation within the active site.

##### Methicillin-Resistant *Staphylococcus aureus* PBP4 with Salvianic Acid A

**Figure 6:**
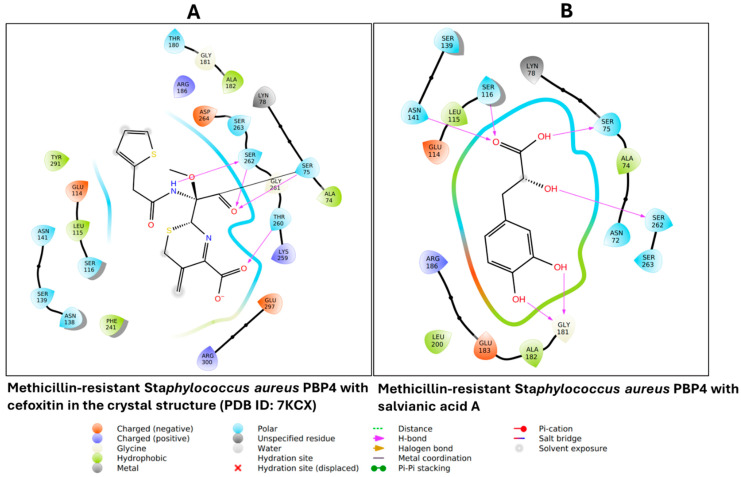
(**A**). Interaction of methicillin-resistant *Staphylococcus aureus* PBP4 with cefoxitin in the crystal structure (PDB ID: 7KCX. (**B**). Methicillin-resistant *Staphylococcus aureus* PBP4 with salvianic acid A.

Cefoxitin exhibited a compact interaction pattern in the active site of *Staphylococcus aureus* PBP4. Cefoxitin was mainly stabilized by hydrogen bonds with residues such as Ser262, Ser75, and Thr260 (Figure 6A). Similarly, salvianic acid A formed hydrogen bonds with common residues (Ser262 and Ser75), Asn141, and Ser116, reflecting enhanced polar stabilization due to its poly-phenolic scaffold and carboxylic group (Figure 6B). Multiple van der Waals interactions and a few hydrophobic contacts may contribute to the inhibition of the protein by blocking the substrate recognition regions of MRSA PBP4 [36,45,46].

##### *Staphylococcus aureus* ATCC D-Ala-D-Ala Ligase with 1-O-Caffeoylquinic Acid

**Figure 7:**
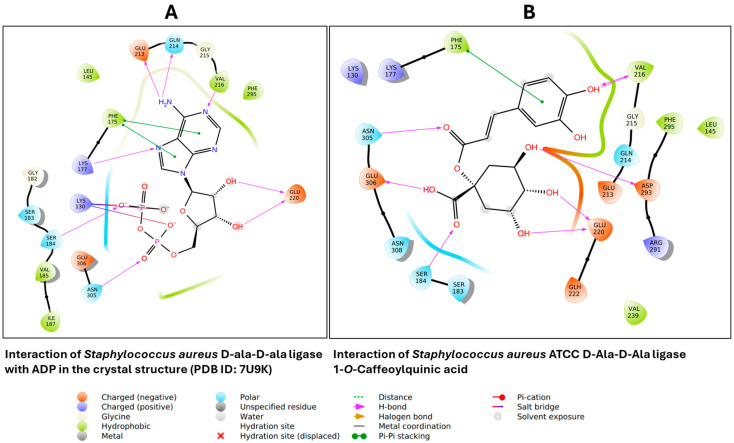
(**A**). Interaction of *Staphylococcus aureus* D-alanine-D-alanine ligase with ADP in the crystal structure (PDB ID: 7U9K). (**B**). Interaction of *Staphylococcus aureus* ATCC D-Ala-D-Ala ligase with 1-*O*-caffeoylquinic acid.

ATP binds within the catalytic pocket of D-Ala-D-Ala ligase (PDB ID: 7U9K) through a coordinated network of hydrogen bonding, electrostatic, and hydrophobic interactions that stabilize the nucleotide in a catalytically competent orientation (Figure 7A). The adenine ring is anchored by hydrogen bonds with Glu213, Gln214, Val216, and Lys177, while π-mediated interactions with Phe175 contribute to stabilization of the purine scaffold. The ribose hydroxyl groups further interact with Glu220, whereas the triphosphate moiety forms strong electrostatic and hydrogen bonding interactions with Lys130, Ser184, and Asn305, residues essential for phosphate coordination and ATP-dependent catalysis. These interactions are consistent with the conserved ATP-recognition mechanism reported for bacterial D-ala-D-ala ligases and other ATP-grasp enzymes involved in peptidoglycan biosynthesis and antibiotic targeting [47].

Similarly, salvianolic acid interacts with *Staphylococcus aureus* ATCC D-Ala-D-Ala ligase through a combination of hydrogen bonding, aromatic contacts, and van der Waals interactions (Figure 7B). Like in the crystal complex, key interactions are observed with catalytically essential residues, including Glu220, Asp293, and Glu306, indicating strong engagement between the ligand oxygen atoms and charged residues within the active site. Additional hydrogen bonding with Ser184 and Asn305, as well as multiple van der Waals interactions with nearby residues, further reinforces ligand stabilization, highlighting the importance of polar interactions in this complex. Such hydrogen bond networks are consistent with known binding patterns in D-Ala-D-Ala ligase systems [48].

Furthermore, a π–π stacking interaction with Phe175 helps maintain the ligand’s orientation within the binding pocket. This interaction, together with hydrophobic contacts from surrounding hydrophobic residues such as Phe295 and Val239, contributes to a stable and well-packed binding environment [49].

## 3. Materials and Methods

### 3.1. Plant Material and Extract Preparation

*Clinopodium albanicum* (Griseb. ex K. Malý) Melnikov (*Lamiaceae*) was collected from Prizrenska Bistrica during the flowering period in July 2020. Aerial parts were harvested from 15 individual plants, pooled into a composite sample, and stored under deep-freezing conditions (−80 °C). Prior to hydrodistillation, the material was subsequently freeze-dried. Following hydrodistillation of the essential oil using a Clevenger apparatus (Faculty of Chemistry, Belgrade, Serbia), (100 g of plant material and 2 L of distilled water), the remaining liquid residue was collected and lyophilized to obtain a dry solid extract.

The dry solid extract of *C. albanicum* (20 mg) was diluted with 1 mL of 96% methanol (w:v = 10:1), vortexed and sonicated for 20 min in an ultrasonic bath (RK100, Bandelin, Berlin, Germany) for chemical analysis and antioxidant assay. After centrifugation at 10,000 *g* for 10 min, the supernatant was filtered through cellulose filters with a pore size of 0.2 µm (Agilent Technologies, Santa Clara, CA, USA) and stored at 4 °C until analyses. For antimicrobial assays, dry solid extract was dissolved in 30% ethanol (10 mg/mL).

### 3.2. UHPLC(−)HESI–QqQ-MS/MS Untargeted Metabolomics Analysis

The extract’s metabolic profile was ascertained using the Liquid Chromatography–High-Resolution Tandem Mass Spectrometry (LC-HRMS/MS, Thermo Scientific™ Vanquish™ Core HPLC system, Thermo Fisher Scientific, Waltham, MA, USA) connected to the Orbitrap Exploris 120 mass spectrometer, San Jose, CA, USA. The Hypersil GOLDTM C18 analytical column (50 × 2.1 mm, 1.9 μm particle size) was part of the liquid chromatography system. The flow rate remained steady at 300 μL/min, and the injection volume was 5 μL. Ultrapure water + 0.1% formic acid (A) and acetonitrile (MS grade) + 0.1% formic acid (B) were used to elute the compounds of interest: 5% B for the first minute, 5–95% B for 1–10 min, 95% B for 10–12 min, and 5% B for 15 min.

An ESI source running in negative ionization mode was installed in the Orbitrap Exploris 120 mass spectrometer (Thermo Fisher Scientific, Waltham, MA, USA). While data-dependent MS2 tests were carried out at an Orbitrap resolution of 15,000 FWHM with the normalized collision energy for CID set to 35%, full-scan MS were monitored from 100 to 1500 *m*/*z* at an Orbitrap resolution of 60,000 FWHM. The intensity threshold was set to 1 × 10^5^, and the dynamic exclusion duration was set to 10 s, with exclusion from a particular scan after two occurrences. Based on their chromatographic behavior, MS and MS2 comparisons with standard compounds, when available, and literature data that offered a preliminary identification, the phenolic compounds were identified. Data acquisition was carried out with the Xcalibur^®^ data system (Thermo Finnigan, San Jose, CA, USA) [50].

### 3.3. Antioxidant Activity Assays

The antioxidant capacity of the *C. albanicum* by-product was evaluated by spectrophotometric methods, including 2,2-diphenyl-1-picrylhydrazyl (DPPH) radical scavenging activity, 2,2′-azino-bis-(3-ethylbenzothiazoline-6-sulfonic acid) (ABTS) radical scavenging activity and ferric ion-reducing antioxidant power (FRAP) assays. All measurements were performed in triplicate.

The DPPH assay was performed according to the modified method of Brand-Williams et al. [51]. In brief, a 0.2 mM DPPH stock solution was prepared by dissolving DPPH in methanol. The reaction mixture (500 µL DPPH solution, 450 µL methanol and 50 µL sample/reference compound) was incubated for 30 min in the dark at room temperature. The absorbance was measured at 517 nm with an Agilent 8453 spectrophotometer (Agilent Technologies, Waldbronn, Germany). The percentage of radical scavenging activity was calculated using the following equation: DPPH radical scavenging activity (%) = [(A_control_ − A_sample_)/A_control_] × 100, where A_sample_ is the absorbance of the reference compound/sample reaction mixture and A_control_ is the absorbance of the DPPH solution. The calibration line was established using methanol solutions of gallic acid (GA), and the results are expressed as mmol GA equivalents per 100 mg^−1^ extract.

The ABTS assay was done according to the method of Re et al. [52] with some modifications [53]. The stock solutions included a 7 mM ABTS solution and a 2.45 mM potassium persulfate solution (final concentration). The working solution was generated by mixing the two stock solutions in equal volumes and incubating the mixture at room temperature in the dark (12–16 h). The ABTS radical cation (ABTS^•+^) solution was diluted with 80% ethanol and equilibrated at 30 °C to obtain an absorbance of 0.700 ± 0.02 nm at 734 nm. The reaction mixture was prepared by mixing 970 µL of the ABTS^•+^ solution with 30 µL of a plant methanol extract or standard solution and then incubating it for 10 min at room temperature in the dark. The percentage scavenging activity of the decoction against ABTS^•+^ was calculated as % inhibition of absorbance at 734 nm using the following formula: [(A_control_ − A_sample_)/A_control_] × 100, where A_sample_ is the absorbance of the reference compound/ tested extract solution and A_control_ is the absorbance of the ABTS^•+^ solution. GA was used as the reference compound, and the results are expressed as mmol GA equivalents per 100 mg^−1^ extract.

The FRAP assay was conducted as previously described by Benzie and Strain [54] with slight modifications. The FRAP working reagent was prepared by mixing 300 mM Na-acetate buffer (pH = 3.6), 10 mM 2,4,6-tri(2-pyridil)-1,3,5-triazine (TPTZ) dissolved in 40 mM HCl solution and 20 mM FeCl_3_ in a 10:1:1 ratio immediately before the assay. Aliquots of 50 µL of the methanolic solution of the reference compound (GA)/sample were mixed with 950 µL of FRAP reagent. The resulting mixture was incubated for 10 min at room temperature. The absorbance of the reaction mixture was measured at 593 nm. The blank was prepared by substituting the same amount of diluted extract with methanol. The results are expressed as GA equivalents reducing activity (mmol GAE per 100 mg^−1^ extract).

### 3.4. Antimicrobial Activity Assays

The antimicrobial activity of the optimized extract was evaluated using the broth microdilution method previously described [43]. The assay was performed against a panel of clinically relevant Gram-positive and Gram-negative bacteria, including *Staphylococcus aureus* (ATCC 11632), *Staphylococcus aureus* (IBRS MRSA 011), *Listeria monocytogenes* (ATCC 35152), *Listeria monocytogenes* (ATCC 19111), *Pseudomonas aeruginosa* (IBRS P001), *Pseudomonas aeruginosa* (ATCC 27853), *Salmonella enterica* serovar Typhimurium (ATCC 13311), and *Escherichia coli* (ATCC 25922).

The antifungal activity was assessed against the opportunistic pathogenic yeasts *Candida auris* (CDC B 11903) and *Candida parapsilosis* (ATCC 22019).

The antimicrobial efficacy of the samples was determined by establishing the minimum inhibitory concentration (MIC), minimum bactericidal concentration (MBC), and minimum fungicidal concentration (MFC), expressed in mg/mL. The assays were performed in triplicate using twofold serial dilutions. Negative controls included the solvent used for extract preparation (water) and 30% EtOH used for dissolving the dry extract. Antibacterial activity was assessed against the standard agents streptomycin and ampicillin, while ketoconazole and bifonazole were used as reference antifungal compounds.

### 3.5. Antibiofilm Activity Assay

The impact of the extracts on the *P. aeruginosa* biofilms was determined as described previously in Bukvicki et al., [26] with some modifications. In order to establish biofilms, *P. aeruginosa* was incubated in Triptic soy broth with 2% glucose for 24 h at 37 °C in 96 well microtiter plates with adhesive bottoms (Sarstedt, Nümbrecht, Germany). After incubation, wells were washed twice with sterile PBS, and the biofilms were treated with 2xMBC, MBC and MIC of the extracts for another 24 h at 37 °C. Afterwards, each well was washed twice with PBS, and the biofilms were fixed with methanol. The plates were then air-dried, and the biofilms were stained with 0.1% crystal violet (Bio-Merieux, Marcy-l′Étoile, France) for 30 min. Later, the crystal violet was removed, and the wells were washed with water and air-dried. Bound dye was dissolved in 100 μL of 96% ethanol (Zorka, Novi Sad, Serbia). The absorbance was read at 620 nm on a Multiskan™ FC Microplate Photometer (Thermo Scientific™), and the percentage of biofilm destruction was calculated by the formula: [(A620 control − A620 sample)/A620 control] × 100.

### 3.6. In Silico Analysis

A multi-target molecular docking strategy was employed to evaluate the antibacterial potential of the studied compounds by focusing on key enzymes involved in essential bacterial metabolic pathways. The selected targets included penicillin-binding protein 4 (PBP4) [41,55] and D-alanine–D-alanine ligase, which are critical for cell wall biosynthesis [56]; enoyl-acyl carrier protein reductase (FabI) involved in fatty acid synthesis [39]; and dihydrofolate reductase (DHFR), which plays an essential role in folate metabolism and nucleotide biosynthesis [57]. These proteins are well-established antibacterial drug targets, possessing experimentally determined ligand-bound crystal structures suitable for structure-based docking analyses. Together, these targets represent four fundamental bacterial pathways: cell wall biosynthesis, fatty acid synthesis, DNA replication, and folate metabolism. Therefore, they provide a comprehensive framework for evaluating the potential antibacterial mechanisms of the investigated compounds (Table 6).

The X-ray crystal structures of the selected microbial targets were retrieved from the Protein Data Bank [58]. Protein preparation was performed using the PrepareProtein module in PlayMolecule (https://www.playmolecule.com/) (accessed on 15 March 2026), in which missing hydrogen atoms were added and protonation states of ionizable residues were assigned based on predicted pKa values at physiological pH (7.4). Ligand structures were obtained from the PubChem database and subjected to energy minimization in UCSF Chimera to obtain stable conformations [59].

Docking simulations were performed using AutoDock 4.2.6 [60]. Input files for both proteins and ligands were prepared in MGLTools 1.5.6 by assigning Gasteiger charges and merging nonpolar hydrogens. The grid box was centered on the co-crystallized ligand within each protein, with dimensions of 45 × 45 × 45 Å to encompass the active site. Ligand sampling was carried out using the Lamarckian Genetic Algorithm with 10 independent runs per complex. Docked poses were ranked by binding energy, and the top conformations were chosen for interaction analysis and visualization with Maestro’s Protein–Ligand Interaction Viewer 2019-2.

## 4. Conclusions

The present study provides the first comprehensive insight into the chemical composition and biological potential of the by-product obtained after hydrodistillation of *Clinopodium albanicum*, an endemic Balkan species. The results clearly demonstrate that this post-distillation by-product is not an inert waste material but rather a chemically rich and biologically active matrix with significant valorization potential. Untargeted LC–HRMS/MS metabolomics revealed a highly complex phytochemical profile dominated by hydroxycinnamic acid derivatives, particularly rosmarinic acid and structurally related caffeic acid conjugates such as sagerinic and salvianolic acids. This phenylpropanoid-driven metabolic signature, complemented by a diverse flavonoid spectrum (both glycosylated and methoxylated forms), firmly positions the extract within a characteristic *Lamiaceae* phenolic framework. Importantly, the simultaneous presence of primary metabolites (sugars, organic acids) and oxylipin-related compounds indicates that the extract retains a broad metabolic background, which may synergistically contribute to its overall functionality. The pronounced antioxidant activity observed across DPPH, ABTS, and FRAP assays is consistent with the identified phenolic composition, particularly the abundance of rosmarinic acid and flavonoids, confirming that these compounds remain preserved in the by-product after essential oil extraction. In parallel, the extract exhibited notable antibacterial activity against both Gram-positive and Gram-negative bacteria, including clinically relevant and resistant strains, as well as significant antifungal effects against *Candida* species. The strong antibiofilm activity against *Pseudomonas aeruginosa*, even at subinhibitory concentrations, further highlights its potential in targeting microbial virulence mechanisms. Molecular docking analysis supported these findings by demonstrating favorable binding interactions of major phytochemicals with key bacterial targets involved in cell wall biosynthesis, fatty acid metabolism, and nucleotide synthesis. The particularly strong affinities observed for salvianolic acid, as well as quercetin derivatives, suggest that multiple mechanisms of antibacterial action may be involved, reinforcing the concept of a multi-target natural antimicrobial system.

This study highlights that the hydrodistillation by-product of *C. albanicum* represents a valuable and sustainable source of bioactive compounds with pronounced antioxidant, antimicrobial, antifungal, and antibiofilm properties. These findings strongly support its further exploration within circular economy frameworks, particularly for applications in nutraceuticals, functional foods, pharmaceuticals, and biomaterials. Moreover, given the endemic nature of the species, the identified unique phytochemical profile may offer additional opportunities for the development of region-specific, high-value natural products.

## Figures and Tables

**Table 1:** Identified compound names, retention times (Rt, min), molecular formulas, MS data (calculated and exact masses, as well as mean mass accuracy—Δ mDa), major MS^2^ fragment ions of the compounds present in the extract of *Clinopodium albanicum* (Griseb. ex K.Malý) Melnikov.

No	Compound Name	*t*_R_, min	Molecular Formula, [M–H]^–^	Calculated mass, *m/z*	Exact Mass, *m/z*	Δ ppm	MS^2^ Fragments, (% Base Peak)	Peak Area	Ref
*Hydroxybenzoic acids*									
1	Hydroxybenzoic acid hexoside	0.68	C13H15O8–	299.07724	299.07752	−0.92	59.01383(1), 71.0137(1), 89.02441(1), 93.03463(33), 137.02452(100), 138.02823(1)	7030282	[17]
2	Syringic acid hexoside	0.72	C15H19O10–	359.09837	359.09827	0.29	72.99316(19), 179.03516(20), **197.04568**(100), 359.09854(11)	143509389	[17]
3	Vannilic acid hexoside	0.74	C14H17O9–	329.08781	329.08806	−0.77	108.02175(13), 123.04522(32), 152.01161(18), **167.03503**(100)	34216505	NA
4	Gallic acid hexoside	0.76	C13H15O10–	331.06707	331.06751	−1.32	125.02457(57), 149.99644(4), 168.00664(92), 169.01428(15), **331.06757**(100)	12884000	[17]
5	Dihydroxybenzoic acid hexoside	0.80	C13H15O9–	315.07216	315.07242	−0.85	108.02176(41), 109.02964(34), 123.04526(11), **152.01170**(100), 153.01964(61), 315.07251(47)	98539890	[17]
6	Dihydroxybenzoic acid	0.96	C7H5O4–	153.01933	153.01950	−1.12	**109.02965**(100), 153.01950(33)	12703337	[18]
7	Vannilic acid	5.51	C8H7O4–	167.03498	167.03513	−0.88	108.02181(38), 152.01134(26), **167.03520**(100)	2800888	[18]
8	Hydroxybenzoic acid	6.26	C7H5O3–	137.02442	137.02455	−0.97	**93.03467**(100), 137.02461(71)	19435740	[18]
*Caffeoylquinic acids*									
9	1-*O*-caffeoylquinic acid	1.17	C16H17O9–	353.08781	353.08776	0.14	135.04532(29), 173.04579(17), 179.03514(80), **191.05630**(100)	171404226	[17]
10	Caffeic acid hexoside	4.42	C15H17O9–	341.08781	341.08779	0.03	135.04532(20), **179.03520**(100)	11200865	[17]
11	*P*-Coumaric acid	4.70	C9H7O3–	163.04007	163.04018	−0.71	119.0504(37), 163.04013(100)	10759062	[19]
12	Caffeic acid	4.70	C9H7O4–	179.03498	179.03510	−0.66	**135.04532**(100), 137.03577(7), 179.03525(23)	75518084	[19]
13	Coumaric acid hexoside	4.70	C15H17O8–	325.09289	325.09312	−0.69	119.05032(52), 145.02962(3), **163.04021**(100)	26470043	NA
14	4-*O*-caffeoylquinic acid	4.83	C16H17O9–	353.08781	353.08767	0.39	93.03471(6), 135.04521(33), **173.04559**(100), 179.03502(72), 191.05618(53)	1055827424	[17]
15	Caffeoyltronic acid	5.25	C13H13O8–	297.06159	297.06180	−0.71	75.00881(5), **135.03008**(100), 161.02449(4), 179.03517(7)	58536059	[17]
16	4-*O*-Coumaroylquinic acid	5.32	C16H17O8–	337.09289	337.09316	−0.81	93.03461(7), 119.05022(7), 163.04033(24), **173.04567**(100), 191.05598(5)	18199474	[17]
17	Sinapic acid hexoside	5.34	C17H21O10–	385.11402	385.11417	−0.37	101.02454(7), 153.09233(7), **161.02463**(100), 208.03824(6), 223.06087(8), 385.11414(21)	4611276	[17]
18	Caffeoylshikimic acid	5.45	C16H15O8–	335.07724	335.07758	−1.01	135.04535(26), **161.02449**(100), 179.03487(24)	6115696	[17]
19	4-*O*-Feruloylquinic acid	5.53	C17H19O9–	367.10346	367.10356	−0.27	93.03476(13), 134.03734(14), 161.02438(92), **173.04590**(100), 191.05469(7), 193.05119(15)	6199734	[17]
20	Yunnaneic acid E	5.69	C27H23O14–	571.10933	571.10958	−0.44	72.99315(23), 109.02955(30), **135.04520**(100), 179.03506(37), 197.04564(65)	64081123	NA
21	Lithospermic acid	5.71	C27H21O12–	537.10385	537.10392	−0.14	133.03003(10), 135.04506(30), **161.02472**(100), 179.03542(63), 281.06790(13), 295.06110(91)	8243505	[17]
22	Salvianolic acid K	5.83	C27H23O13–	555.11442	555.11462	−0.37	283.06143(92), 295.06146(38), 313.07184(29), 331.08276(20), 339.05078(45), **357.06165**(100)	203024744	NA
23	Yunnaneic acid D	5.91	C27H23O12–	539.11950	539.11964	−0.26	**135.04532**(100), 179.03519(58), 197.04568(69), 253.08722(53), 271.09796(71), 315.08774(31)	27143786	[17]
24	Yunnaneic acid F	5.92	C29H25O14–	597.12498	597.12525	−0.45	**135.04530**(100), 179.03522(40), 197.04576(77), 267.10291(82), 293.08224(31), 311.09286(32)	64734062	[17]
25	Bis-caffeic acid hexoside	6.13	C24H23O12–	503.11950	503.11970	−0.40	89.02451(26), 119.03517(11), 135.04532(37), 161.02472(45), **179.03534**(100), 323.07761(16)	4411276	[17]
26	Methyl yunnaneate E	6.13	C28H25O14–	585.12498	585.12534	−0.62	72.99314(22), **135.04529**(100), 179.03513(34), 197.04572(45), 273.11353(13), 299.09286(21)	74790782	NA
27	Methylrosmarinic acid hexoside	6.28	C25H27O13–	535.14572	535.14614	−0.79	179.03532(22), **197.04582**(100), 323.07648(3), 337.09552(5), 359.09848(45)	6342257	NA
28	Rosmarinic acid	6.39	C18H15O8–	359.07724	359.07716	0.21	72.99313(15), 135.04532(7), **161.02455**(100), 179.03511(20), 197.04567(35)	2501321749	[17]
29	Sagerinic acid	6.42	C36H31O16–	719.16176	719.16157	0.27	**161.02460**(100), 179.03545(14), 197.04572(47), 271.09796(8), 297.07755(13), 359.07315(6)	770561914	[17]
30	Ferulic acid	6.46	C10H9O4–	193.05063	193.05072	−0.45	134.03752(26), 161.02455(18), 178.02753(6), **193.05074**(100)	10185616	[18]
*Flavonoid glycosides*									
31	Quercetin 3,7-di-*O*-hexoside	5.02	C27H29O17–	625.14102	625.14151	−0.78	299.02023(69), 300.02847(14), 301.03540(54), **462.08109**(100), 463.08694(34)	4593594	[17]
32	Apigenin 6,8-di-*C*-hexoside	5.34	C27H29O15–	593.15119	593.15141	−0.36	**353.06656**(100), 383.07733(66), 413.08844(9), 473.10925(49), 503.11972(17), 593.15143(33)	30124218	[17]
33	Quercetin 3-*O*-(6″-rhamnosyl)-hexoside	5.85	C27H29O16–	609.14611	609.14648	−0.61	151.00389(3), 178.99886(3), **300.02762**(100), 301.03522(45), 609.14642(14)	569102085	[17]
34	Quercetin 3-*O*-hexoside	5.96	C21H19O12–	463.08820	463.08835	−0.32	151.00423(3), 178.99890(3), **300.02783**(100), 301.03546(37)	415571504	[17]
35	Kaempferol 3-*O*-(6″-rhamnosyl)-hexoside	6.09	C27H29O15–	593.15119	593.15168	−0.82	284.03305(43), **285.03903**(100)	115764918	[17]
36	Apigenin 7-*O*-(6″-rhamnosyl)-hexoside	6.18	C27H29O14–	577.15628	577.15671	−0.75	**269.04575**(100)	32285411	[17]
37	Kaempferol 3-*O*-hexoside	6.20	C21H19O11–	447.09329	447.09353	−0.56	227.03516(4), 255.03023(11), **284.03293**(100), 285.04059(32), 447.0939(20)	181335136	[17]
38	Quercetin 3-*O*-(6″-acetyl)-hexoside	6.28	C23H21O13–	505.09877	505.09929	−1.03	**300.02783**(100), 301.03522(26)	10644816	NA
39	Diosmetin 7-*O*-(6″-rhamnosyl)-hexoside	6.29	C28H31O15–	607.16684	607.16740	−0.91	284.03290(18), **299.05634**(100)	175528492	[17]
40	Apigenin 7-*O*-hexoside	6.32	C21H19O10–	431.09837	431.09839	−0.04	268.03802(93), 269.04590(52), **431.09869**(100)	8740486	[19]
41	Kaempferol 3-*O*-(6″-acetyl)-hexoside	6.58	C23H21O12–	489.10385	489.10422	−0.75	255.03011(16), **284.03284**(100), 285.04053(20), 489.10370(16)	4977631	[19]
42	Acacetin 7-*O*-(6″-rhamnosyl)-hexoside	6.90	C28H31O14–	591.17193	591.17208	−0.26	268.03790(9), **283.06128**(100)	89184055	[17]
*Flavonoid aglycones*									
43	Luteolin	6.98	C15H9O6–	285.04046	285.04067	−0.73	151.00441(3), 175.0405(3), **285.04062**(100)	4763134	[17]
44	Quercetin	6.98	C15H9O7–	301.03538	301.03570	−1.07	107.01365(4), 121.02944(18), **151.00389**(100), 178.99898(51), 273.04037(6), 301.03568(72)	10249953	[17]
45	Trihydroxy-dimethoxyflavone isomer 1	7.14	C17H13O7–	329.06668	329.06711	−1.31	299.02008(23), **314.04340**(100), 329.06610(16)	21218762	[17]
46	Trihydroxy-dimethoxyflavone isomer 2	7.29	C17H13O7–	329.06668	329.06709	−1.26	**299.01999**(100), 314.04355(93), 315.04697(3), 329.06720(14)	68943078	[17]
47	Naringenin	7.30	C15H11O5–	271.06120	271.06151	−1.16	107.0134(13), 119.0503(36), **151.00385**(100), 177.01987(13), 271.06155(56)	7331471	[17]
48	Apigenin	7.35	C15H9O5–	269.04555	269.04587	−1.19	149.02460(3), 151.00389(3), 225.05554(3), **269.04581**(100)	9924070	[17]
49	Trihydroxy-trimethoxyflavone isomer 1	7.39	C18H15O8–	359.07724	359.07732	−0.22	**329.03064**(100), 344.05380(90), 359.07748(8)	386626681	[17]
50	Kaempferol	7.41	C15H9O6–	285.04046	285.04077	−1.08	151.00327(3), **285.0408**(100)	6609040	[17]
51	Trihydroxy-trimethoxyflavone isomer 2	7.77	C18H15O8–	359.07724	359.07736	−0.33	**329.03061**(100), 344.05368(51), 359.07764(10)	152992129	[17]
52	Diosmetin	7.94	C16H11O6–	299.05611	299.05652	−1.37	271.09781(19), 284.03308(59), **299.05640**(100)	46973896	[17]
53	Quercetin 3′,4′,7-trimethyl ether	8.23	C18H15O7–	343.08233	343.08244	−0.34	298.01239(5), **313.03583**(100), 314.03925(3), 328.05942(40), 343.08295(6)	62841872	[20]
54	Chrysin	8.30	C15H9O4–	253.05063	253.05100	−1.47	209.06133(3), 253.05096(100)	1435551	[18]
55	Dihydroxy-tetramethoxyflavone	8.32	C19H17O8–	373.09289	373.09308	−0.50	328.02380(11), **343.04587**(100), 358.06955(58), 373.17908(8)	31239014	[17]
56	Acacetin	8.40	C16H11O5–	283.06120	283.06156	−1.27	268.03793(84), **283.06137**(100)	185015740	[13]
*Other metabolites*									
57	Tuliposide B	0.50	C11H17O9–	293.08781	293.08801	−0.71	73.02946(50), 129.01953(41), 131.04616(92), **159.03006**(100), 245.06673(49)	25552279	[17]
58	Sucrose	0.50	C12H21O11–	341.10894	341.10877	0.49	71.01389(37), **89.02445**(100), 101.02449(30), 113.02454(24), 119.03506(35), 179.05618(7)	1099455915	[17]
59	Hexose	0.51	C6H11O6–	179.05611	179.05626	−0.81	**59.01388**(100), 71.01393(47), 85.02961(5), 89.02451(44), 101.02455(6), 113.02464(6)	205462486	NA
60	Quinic acid	0.51	C7H11O6–	191.05614	191.05628	−0.72	85.02959(7), 127.04015(3), **191.05627**(100)	731448971	[17]
61	Malic acid	0.53	C4H5O5–	133.01420	133.01435	−1.11	71.01390(36), 72.99316(9), 89.02450(7), **115.00379**(100), 133.01439(47)	484992938	[17]
62	Maleic acid	0.54	C4H3O4–	115.00370	115.00389	−1.64	**71.01389**(100), 115.00388(17)	86339713	NA
63	Salvianic acid A	0.82	C9H9O5–	197.04553	197.04585	−1.64	**72.99317**(100), 123.04527(53), 135.04530(72), 179.03517(48), 197.04585(9)	658013124	NA
64	Tuberonic acid hexoside	5.28	C18H27O9–	387.16606	387.16602	0.10	**59.01387**(100), 89.02447(33), 101.02455(19), 119.03515(18), 207.10284(28), 387.16656(47)	1525550306	NA
65	Benzoyl-glyceryl hexuronic acid	5.53	C16H19O10–	371.09837	371.09852	−0.40	75.00887(14), 85.02952(15), 113.02457(26), **121.02967**(100), 161.02451(8), 249.06197(54)	59749738	NA
66	Vinylphenyl-pentosyl-hexoside	5.78	C19H25O10–	413.14532	413.14535	−0.08	**57.03461**(100), 99.04519(87), 101.02448(28), 125.0245(31), 161.02487(47), 269.10257(14)	68833284	NA
67	Syringaresinol hexoside	6.23	C28H35O13–	579.20832	579.20811	0.35	166.02751(10), **181.05084**(100), 269.04590(41), 271.05286(35), 402.13242(25), 417.15555(32)	2753185	[17]
68	Trihydroxy-octadecadienoic acid	7.49	C18H31O5–	327.21770	327.21811	−1.27	85.02953(63), 183.13913(86), 211.13419(31), 251.16544(28), **327.21805**(100)	246143102	[17]
69	Linoleic acid hydroperoxide	8.59	C18H29O4–	309.20713	309.20767	−1.75	113.09735(70), 171.10280(63), **195.10278**(100), 221.15480(77), 251.16559(20), 291.19696(63)	54082610	[17]

NA—Not applicable.

**Table 2:** Antioxidant activity of the methanolic extract of *Clinopodium albanicum* (Griseb. ex K.Malý) Melnikov by-product as determined by DPPH, ABTS, and FRAP assays (mean ± SE).

	DPPH mmol GAE per 100 mg Extract	ABTS mmol GAE per 100 mg Extract	FRAP mmol GAE per 100 mg Extract
*C. albanicum* by-product	32.54 ± 1.28	27.80 ± 0.66	35.95 ± 0.77

GAE—gallic acid equivalents.

**Table 3:** Antibacterial activity of the post-distillation residue of *Clinopodium albanicum* (Griseb. ex K.Malý) Melnikov and ampicillin against Gram-positive and Gram-negative bacteria, expressed as minimum inhibitory concentration (MIC) and minimum bactericidal concentration (MBC) values (mg/mL).

Strain	*C. albanicum*		Ampicillin	
	MIC	MBC	MIC	MBC
*Staphylococcus aureus* (ATCC 11632)	1.0	2.0	0.00630	0.01300
*Staphylococcus aureus* (IBRS MRSA 011)	1.0	2.0	0.00160	0.00320
*Listeria monocytogenes* (ATCC 35152)	0.5	1.0	0.00014	0.00028
*Listeria monocytogenes* (ATCC 19111)	0.5	1.0	0.00014	0.00028
*Pseudomonas aeruginosa* (IBRS P001)	1.0	2.0	0.01250	0.02500
*Pseudomonas aeruginosa* (ATCC 27853)	1.0	2.0	0.00040	0.00080
*Salmonela enterica* (ATCC 13311)	1.0	2.0	0.00160	0.00320
*Escherichia coli* (ATCC 25922)	0.5	1.0	0.00160	0.00320

**Table 4:** Antifungal activity of the post-distillation residue of *Clinopodium albanicum* (Griseb. ex K.Malý) Melnikov and Intraconazole against *Candida* species, expressed as minimum inhibitory concentration (MIC) and minimum fungicidal concentration (MFC) values (mg/mL).

Strain	*C. albanicum*		Itraconazole	
	MIC	MFC	MIC	MFC
*Candida auris* (CDC B 11903)	0.50	1.00	0.003	0.0060
*Candida parapsilosis* (ATCC 22019)	0.25	0.50	0.006	0.0012

**Table 5:** Inhibitory effect of the post-distillation residue of *Clinopodium albanicum* (Griseb. ex K.Malý) Melnikov on *Pseudomonas aeruginosa* biofilm formation, expressed as the percentage (%) of biofilm inhibition relative to the untreated control.

	MIC	½ MIC	¼ MIC	⅛ MIC
*C. albanicum* by-product	95.52	86.82	83.86	40.65

**Table 6:** Microbial target proteins and available 3D structures suitable for docking calculations.

Bacterium	Target Protein	Example PDB	Ligand	Ref
*Staphylococcus aureus*/MRSA	PBP4	7KCX	Cefoxitin	[55]
*Listeria monocytogenes*	PBP4	3ZG8	Ampicillin	[41]
*Staphylococcus aureus* ATCC	D-Ala-D-Ala ligase	7U9K	ATP	[56]
*Pseudomonas aeruginosa*	FabI	4NR0	Triclosan	[39]
*Escherichia coli*	DHFR	6CQA	AMQPD	[57]

## Data Availability

Data will be made available upon request from the corresponding author.

## Supplementary Materials

The following supporting information can be downloaded at: https://www.mdpi.com/article/10.3390/plants15111748/s1, Table S1. Validation of the molecular docking protocol by redocking the co-crystallized ligands into the active sites of their corresponding target proteins.
