# Supplementary material for: Bioactive Potential of Post-Distillation Residue of Clinopodium albanicum (Griseb. ex K. Malý) Melnikov: Phytochemical Profiling, Antioxidant and Antimicrobial Activities with Molecular Docking Insights
Source: Plants (Basel). 2026 Jun 4;15(11):1748. doi: 10.3390/plants15111748 (PMC13259260; doi:10.3390/plants15111748)
Supplement: Supplementary file 1 [file plants-15-01748-s001.zip › plants-4305790-supplementary.pdf]

Table S1. Validation of the molecular docking protocol by redocking the co-crystallized ligands into the active sites of their corresponding target proteins.

PDB ID	RMSD (Å)
7KCX – Cefoxitin	1.55
3ZG8 – Ampicillin	1.81
7U9K – ATP	1.97
4NR0 – Triclosan	0.93
6CQA – AMQPD	1.66
